# Noninvasive respiratory support in the perioperative setting: a narrative review

**DOI:** 10.3389/fmed.2024.1364475

**Published:** 2024-04-17

**Authors:** Luigi Vetrugno, Cristian Deana, Nicolas Colaianni-Alfonso, Fabrizio Tritapepe, Carmen Fierro, Salvatore Maurizio Maggiore

**Affiliations:** ^1^Department of Medical, Oral and Biotecnological Science, “G. D’Annunzio” Chieti-Pescara University, Chieti, Italy; ^2^Department of Anesthesiology, Critical Care Medicine and Emergency, SS. Annunziata Hospital, Chieti, Italy; ^3^Department of Anesthesia and Intensive Care, Health Integrated Agency of Friuli Centrale, Udine, Italy; ^4^Respiratory Intermediate Care Unit, Juan A. Fernandez Hospital, Buenos Aires, Argentina; ^5^Department of Innovative Technologies in Medicine & Dentistry, Section of Anesthesia and Intensive Care, SS. Annunziata Hospital, G. D’Annunzio University, Chieti, Italy

**Keywords:** noninvasive ventilation, atelectasis, high-risk patients, postoperative pulmonary complications, obesity, sleep apnea disorders, airway management

## Abstract

The application of preoperative noninvasive respiratory support (NRS) has been expanding with increasing recognition of its potential role in this setting as a physiological optimization for patients with a high risk of developing atelectasis and postoperative pulmonary complications (PPC). The increased availability of high-performance anesthesia ventilator machines providing an easy way for NRS support in patients with reduced lung function should not be under-evaluated. This support can reduce hypoxia, restore lung volumes and theoretically reduce atelectasis formation after general anesthesia. Therapeutic purposes should also be considered in the perioperative setting, such as preoperative NRS to optimize treatment of patients’ pre-existing diseases, e.g., sleep-disordered breathing. Finally, the recent guidelines for airway management suggest preoperative NRS application before anesthesia induction in difficult airway management to prolong the time needed to secure the airway with an orotracheal tube. This narrative review aims to revise all these aspects and to provide some practical notes to maximize the efficacy of perioperative noninvasive respiratory support.

## 1 Introduction

Noninvasive respiratory support (NRS) can be applied in the perioperative setting using continuous positive pressure (CPAP) or pressure support ventilation (PSV), with or without end-expiratory positive pressure (PEEP) (1, 2). The most common devices for this purpose include oro-nasal and facial masks. Helmets are rarely used in this context, and scarce data about using the recently introduced high flow nasal oxygen (HFNO) devices exist to date. HFNO raises oropharyngeal pressures and increases lung volumes, generating a low level of PEEP (3).

In terms of the preoperative period, two settings need to be investigated. More in deep, NRS used inside the operating room, or used outside the operating room but before surgery where it is still is underutilized (4). Preoperative NRS use is a prophylactic strategy to prevent postoperative pulmonary complications or prolong time to desaturation in a patient with predicted difficult airway management. Other perioperative setting uses could be for therapeutic purposes, for example, preoperative NRS to ameliorate pre-existing patients’ disease, such as sleep-breathing disorders, or to treat postoperative pulmonary complications (5). In this last case, NRS can be used as a continuum from pre-to postoperative period (6).

However, to improve respiratory complications after surgery, prehabilitation or patient education also play a key role, including three important aspects: physiotherapy, smoking cessation, and nutritional support. These strategies have been proposed to prevent atelectasis and postoperative pulmonary complication (PPC). Some studies have suggested the use of preoperative inspiratory muscle training before cardiac or major abdominal surgery to prevent postoperative atelectasis and pneumonia. However, the studies are still underpowered and more data are needed. This topic lies outside the scope of this review, and other papers are more focused on that (7).

This narrative review aims to describe the use of perioperative NRS, bearing in mind the patient’s characteristics and the surgical context. The appropriate interface use will be briefly discussed as a comprehensive approach to perioperative NRS management.

## 2 Preoperative NRS inside the operative room

Noninvasive ventilation in the preoperative setting and inside the operative room has been proposed as an effective way to decrease postoperative morbidity and improve postoperative outcomes in patients at increased risk of PPC (8). Considering that PPC has been reported in 5–33% of total surgical patients, with an associated mortality rate at 30 days as high as 20%, reducing the PPC rate is imperative (9). In 2015, the European joint task force introduced guidelines for the perioperative clinical outcomes defining PPC as a composite outcome of the following postoperative conditions: respiratory infection, respiratory failure, pleural effusion, atelectasis, pneumothorax, bronchospasm, aspiration pneumonia (10). Thus, different risk prediction models have been developed to identify patients at higher risk of PPC.

One of the most popular is the ARISCAT score, which was developed to define postoperative respiratory risk of complications in surgical patients in Catalonia. The ARISCAT score is a seven-variable regression model that considers age, preoperative SpO_2_, respiratory infection in the last month before surgery, preoperative anemia, surgical incision site, surgery duration and the urgency of the procedure. The ARISCAT score stratifies patients into low-, intermediate-, and high-risk of PPC (11). Subsequently, a prospective validation study – PERISCOPE investigation – evaluating the ARISCAT score performance in predicting PPC in other European countries, has been undertaken. The PERISCOPE research outcomes showed suboptimal results in some regions and good results in others, with an overall area under the curve of 0.82 (95% CI 0.79–0.85) (12). Although this score has not been used extensively in clinical practice, it works moderately well. In this regard, the recent international expert panel-based consensus recommendations state that a dedicated score should be used for risk evaluation in a surgical population as a strong recommendation to optimize perioperative patient management (9). Therefore, the correct assignment of perioperative respiratory risk through validated scores allows the proper perioperative management of patients to be predefined.

### 2.1 Atelectasis-related pneumonia

Patients at moderate and high-risk of PPC may be particularly challenging in the perioperative setting for two reasons: high inspired oxygen concentration during pre-oxygenation and the reduced lung volume to which these patients are exposed during general anesthesia lead to atelectasis formation in more than 75% of cases, especially in patients receiving a neuromuscular blocking agent (absorption atelectasis) (8). Atelectasis-related pneumonia is a major complication with rates of up to 1.8% of patients undergoing non-cardiac surgery in the American Society of Anesthesiologists status III, 3.5% after cardiac surgery and 25% of cases following major lung resection for cancer (13). Atelectasis occurs in the most dependent parts of the lungs after the first minute after anesthesia induction. While in a healthy subject atelectasis and impaired oxygenation return to a normal status just after extubation, conversely, some studies have shown that atelectasis are still present many hours after surgery in patients at moderate or high risk of PPC (14). The relevance of interventions directed at minimizing atelectasis has recently been emphasized by an international expert panel, which stated, based on the highest quality of evidence, that the “formation of perioperative clinically significant atelectasis” could “be an important risk factor for the development of postoperative pulmonary complications (11).” However, this topic is subject for future research.

### 2.2 Obesity and atelectasis

Obesity is defined as: “abnormal or excessive fat accumulation that presents a health risk” (15). A patient with a body mass index (BMI) ≥30 is considered obese. Obese patients are more prone to develop atelectasis up to 24 h after surgery, as shown in the computed tomography (CT) scan studies (16). In these patients, the interest in NRS application inside the operating room before anesthesia induction is rapidly growing for three reasons. First, the physiological rationale; second, the increased availability of anesthesia ventilator machines performing pressure support ventilation (PSV) and delivering PEEP level accurately; third, the presence of skilled nurses and expert anesthesiologists more sensitive to the perioperative care of obese patients.

Eichenberger et al., investigating 20 obese patients who underwent laparoscopic gastroplasty and 10 nonobese patients who underwent laparoscopic cholecystectomy, found through CT scan that after 24 h the amount of atelectasis remained unchanged in the obese patients, but showed complete reabsorption in nonobese patients (9.7% versus 1.9%, respectively; *p* < 0.01) (16).

Coussa et al. demonstrated a significant reduction in postintubation atelectasis by CT scan in morbidly obese patients pre-oxygenated with 100% oxygen when 10 cm H_2_O of CPAP was applied for 5 min via face mask (17). At the end of the experiment, atelectasis was present in the experimental and control group but it was much more pronounced in the latter (1.7% ± 1.3% versus 10.4% ± 4.8% in respectively, *p* < 0.001). The study also showed that applying CPAP prolonged the duration of non-hypoxic apnea. A recent systematic review and meta-analysis comprehending 29 articles by Carron et al., comprising 768 patients, found that NRS was associated with a significant improvement in oxygenation (*p* < 0.0001) before tracheal intubation compared to standard preoxygenation. Moreover, they also noted that obese patients receiving NRS for 5 min before and following surgery were exposed to fewer PPC and greater P_a_O_2_ values than the standard preoxygenation group (18). Preoperative NRS and alveolar recruitment maneuvre in morbidly obese patients have also been described to improve respiratory function during intubation (18). Futier et al. showed that 66 morbidly obese patients maintained greater end-expiratory lung volume (EELV) and oxygenation during anesthesia induction with NRS for a longer time compared to conventional preoxygenation alone (19). Therefore, existing data suggest that preoperative NRS in obese patients just before and after general anesthesia may help optimize their management and improve the postoperative course.

### 2.3 Preoperative NRS and post-induction hypoxemia

Preoperative NRS may prolong the safe apnea period and reduce post-induction hypoxemia, increasing the “margin of safety” during anesthesia induction. In this regard, current guidelines on airway management in obese patients and in patients with difficult airways support noninvasive respiratory ventilation with nasal oxygenation while securing an orotracheal tube (20). Recent results on this topic were presented by Zhen et al. In their study, 58 patients who required tracheal intubation or the application of a laryngeal mask under general anesthesia were randomly allocated to receive oxygenation using trans nasal humidified rapid-insufflation ventilator exchange (THRIVE), 30 patients (100% oxygen, 30 ~ 70 litres min − 1), or a facemask, 28 patients (100% oxygen, 10 litres min^−1^) during the pre-oxygenation period and apnea time. The apnea time was significantly increased (*p* < 0.01) in the THRIVE group (21). Furthermore, THRIVE provided a better pre-oxygenation effect than a facemask in the elderly without pulmonary dysfunction. With this approach, the hypoxia onset is delayed, and the duration of apnea time without desaturation extended. Therefore, in the case of rapid sequence induction of anesthesia, THRIVE used for pre-oxygenation could safely extend apnea time during prolonged laryngoscopy and intubation. The administration of HFNO in addition to standard preoxygenation and facemask ventilation is now recommended in high-risk patients (22).

## 3 Preoperative NRS outside the operative room

### 3.1 Preoperative NRS in obstructive sleep apnea

Obese patients are often complicated by the coexistence of obstructive sleep apnea (OSA), a serious preoperative condition characterized by recurrent episodes of complete or partial upper airway obstruction (5). Usually, these patients with long-term domiciliary therapy have their portable ventilators. They put on a mask and operate the device themselves, and are the only patients suitable for preoperative NRS in the ward. A multicenter study of 27,000 patients undergoing general and vascular surgery have shown that approximately 10% of these populations had a diagnosis or a suspicion of OSA (23). However, only half of them were currently treated. Literature has also shown that PPC is increased in these patients when CPAP was not preoperatively used, and that they have approximately twofold or threefold increased risk of cardiorespiratory complications, specifically unplanned reintubation and postoperative myocardial infarction (24). In contrast, the use of preoperative CPAP was associated with a reduction in postoperative cardiovascular complications (cardiac arrest and shock) and a reduction in the length of hospital stay. Considering all these results, the Society of Anesthesia and Sleep Medicine Guidelines recommendation suggests having NRS equipment available for perioperative use or having the patient bring their home CPAP equipment to the surgical facility (25). Furthermore, patients should continue to wear their CPAP device at appropriate times during their hospital stay, both preoperatively and postoperatively. However, only 45% of patients with newly diagnosed OSA have been adherent to NRS therapy in the perioperative period, so it is important to recognize and educate them (26). The opportunity to suspect and discover low compliance patients is increased with the expanding new innovative delivery care models, such as enhanced recovery after surgery (ERAS) and the perioperative surgical home (PSH), which aim to improve patient outcomes and increase efficiency (27–29). Considering that ERAS and PSH have extended the anesthesiologist’s role outside the theater as the most appropriate professional to serve as the “perioperative leader” (30), preoperative NRS prescription tailored for obese or OSA patients should be included in a “pro-active” approach strategy for perioperative risk reduction. A recent study about ERAS after bariatric surgery in the morbidly obese, severely obese, super morbidly obese and super-super morbidly obese using evidence-based clinical pathways that assess the effect of BMI on time to ambulate showed that the use of preoperative CPAP was the only significant predictor of “time to ambulate” and discharging readiness (31). Theoretically, an attractive strategy in these cases could be using NRS in the preoperative phase. However, it should be delivered properly in sub-intensive care or Intensive Care Unit (ICU) settings, with qualified staff and close monitoring (Table 1).

**Table 1:** Indications and effects of perioperative NRS.

Perioperative NRS	Effects
Inside operating room	
Obese patients undergoing major surgery	↓ atelectasis formation ↓ PPC
Obese patients to prolong safe apnea period and reduce post-induction hypoxemia	↓ hypoxia ↑ duration of apnea without desaturation
Hypercapnic and hypoxemic patients	↑ PaO_2_ ↓ PaCO_2_ ↑ FRC ↓ atelectasis formation
Outside and inside operating room	
OSA on long-term domiciliary CPAP	↓ PPC ↓ cardiac complications and shock
Preoperative evaluation of patients with OSA without CPAP	↓ PPC ↓ cardiac complications and shock
Lung resection surgery	No evidence Treatment should not be performed indiscriminately
Postoperative NRS	
Thoracic and abdominal surgery	↓ atelectasis formation ↑ PaO_2_ ↓ PaCO_2_ ↓reintubation risk ↓ICU-acquired infection
Esophageal surgery	↑ROX index

Noninvasive ventilation (NRS); OSA obstructive sleep apnoea; PPC, postoperative pulmonary complication; CPAP, continuous positive airway pressure; ROX index, respiratory rate-oxygenation index.

### 3.2 Preoperative NRS in hypoxemic and hypercapnic patients

In patients with hypercapnic and hypoxemic acute respiratory failure, preoperative NRS is recognized as the treatment of choice (32). In a study in which NRS was initiated in outpatient 7 days before surgery and in which patients were acclimatized to NRS with a 1 h period at FiO_2_ 0.21 under the supervision of one of the investigators, results showed a reduction in the immediate postoperative hypoxemia and improved pulmonary function after abdominal surgery (33). However, as stated above, preoperative NRS should be delivered in an appropriate environment, often requiring patients to be transferred to a specific unit, sub-intensive care or intensive care one (34). This practically translates into increased patient anxiety and requires additional staff as well as ICU-beds with increased hospital costs. Considering the physiological benefits of HFNO application in terms of increased oxygenation and decreased CO_2_, and evaluating its simplicity of use, the limitation of using NRS as stated above could be replaced with preoperative HFNO.

In hypoxemic-hypercapnic patients undergoing lung resection surgery, PPC ranges between 19–59%, a very high rate if compared to abdominal surgery (35). Specific risk factors for PPC in this population result from altered ventilatory function (due to reflex inhibition of the phrenic nerve), effects of general anesthesia, postoperative pain, collapse of the distal airways, and of course, the loss of functional parenchyma caused by resection surgery. In a recent study regarding prophylactic use of NRS in lung resection surgery, patients randomly assigned to PSV received 1-h daily treatment with a facial or no treatment 1 week before surgery (35). The study found no significant differences between using prophylactic PSV or not, suggesting that such treatment should not be performed indiscriminately. Scarcity of literature on this subject calls for future research and more careful patient selection for NRS treatment. Finally, although rare, personalized use of NIV before and during locoregional or spinal anesthesia in patients with a reduced respiratory reserve can be applied (36).

### 3.3 Interfaces

**Figure 1:**
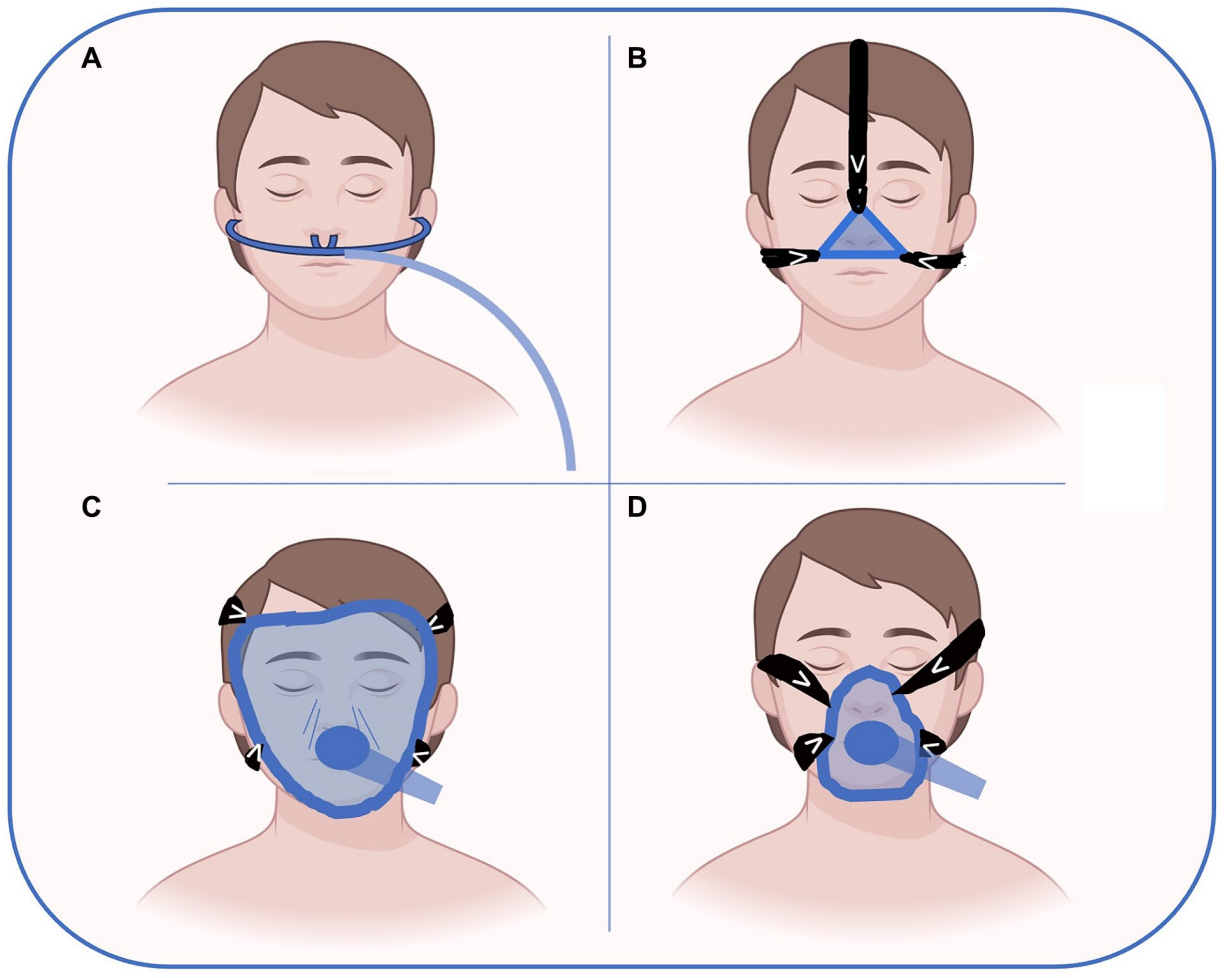
Possible interfaces for preoperative NRS delivery. High flow nasal oxygen **(A)**, nasal CPAP **(B)**, full-face **(C)** and oro-nasal **(D)** masks are the most used interfaces for NRS. In the preoperative setting, nasal devices (**A** or **B**) are usually managed by OSA patients wearing their CPAP at home. The nasal mask **(B)** covers only the nose and gives more comfort than full face or oro-nasal masks. Facial masks **(D)** of different sizes to fit patient face anatomy are the most used interface for preoperative NRS. They are largely available and are the most used during anesthesia induction. Full face masks that cover the entire face are rarely used outside ICU without sedation. Figure has been made with Biorender.com and freely modified by Authors.

Nasal, oro-nasal and full-face masks are the most used interface for NRS. In the preoperative setting, nasal devices are usually managed by OSA patients using their CPAP at home. The nasal mask covers only the nose and gives much comfort. Facial masks of different sizes to fit patients’ face anatomy are the most used interface for preoperative NRS. They are largely available and are the most used during anesthesia induction. Full face masks that cover the entire face are rarely used outside ICU without sedation. It is important to note that all these devices can create damage to the skin and soft tissues as a complication related to the pressure generated to seal the interface as shown in Figure 1. Helmets cover the entire head of the patient with a pneumatic seal at the neck. The helmet is rarely used in the context of preoperative NRS (37).

HFNO delivers warmed, humidified oxygen with flow rates up to 60 litres min^−1^ with FiO_2_ > 90% oxygen, generating also a low level of PEEP. Compared with the use of facemasks, patients refer better comfort. HFNO requires only an oxygen source and can be used in the ward under the supervision of a trained nurse (38). HFNO is becoming very popular in the years since the COVID-19 pandemic, when a large part of hospital resources was been diverted for the respiratory support of infected patients (39). Apart from its current use as suggested by guidelines on airway management in obese patients with difficult airways, there are not yet reported studies in the preoperative setting, for example, to optimize hypoxemic and/or hypercapnic patients before major surgery. To achieve NRS success, you could say that ‘the devil is in detail’, and choosing the correct interface may become challenging. In this regard, seeking a patient-centered perspective is always advisable (40).

## 4 Postoperative noninvasive respiratory support

Patients undergoing intrathoracic or intraabdominal surgery are particularly exposed to PPC (pneumonia, atelectasis, ARDS and respiratory failure) as the result of anesthesia and surgical-related factors. The use of postoperative noninvasive ventilatory support has been investigated as a strategy to prevent PPC. The rationale is that general anesthesia can cause atelectasis and pulmonary collapse, leading to hypoxia through a mismatch of the ventilation/pulmonary perfusion ratio, while surgery induces tissue injury, inflammation, and pain that impair respiratory function and the ability to cough effectively (14). So far, the use of postoperative noninvasive respiratory support has been investigated in depth by two meta-analyses and two large randomized trials. Zayed et al., including 1,865 high-risk patients from 9 studies pairwise meta-analysis, and comparing the outcomes of NRS, HFNO and standard oxygen. They found that NRS was associated with a significant reduction in intubation rate (OR 0.23; 95% CrIs 0.10–0.46), mortality (OR 0.45; 95% CrIs 0.27–0.71), and ICU-acquired infections (OR 0.43, 95% CrIs 0.25–0.70), while high flow nasal cannula (HFNC) was associated with a significant reduction in intubation rate (OR 0.28, 95% CrIs 0.08–0.76) and ICU-acquired infections (OR 0.41; 95% CrIs 0.20–0.80), but mortality was not affected (OR 0.58; 95% CrIs 0.26–1.22) (41). The subgroup study analysis also showed a mortality benefit with NRS over standard oxygen in patients undergoing cardiothoracic surgeries. Furthermore, compared with standard oxygen cardiothoracic patients, NRS and HFNO were associated with lower intubation rates, while NRS reduced the intubation rate only after abdominal surgeries.

Hui S et al., in a recent systematic review and meta-analysis of 38 RCTs including 9,782 adult patients, compared the routine use of CPAP, NRS or HFNO with standard postoperative care (42). They did not observe a difference in postoperative pneumonia frequency between groups (4.9% vs. 5.5%, *p* = 0.23). Postoperative pulmonary complications occurred in 28% of patients receiving noninvasive respiratory support compared with 31% receiving standard care (reduction risk [RD] -0.11 [−0.23 to 0.01]; *I*^2^ = 79%; *p* = 0.07).

In the specific setting of esophageal surgery, we recently reported the benefit of HFNO in a cohort of 71 patients (43). HFNO improved the Respiratory Rate Oxygenation Index (ROX index) after esophagectomy through significant respiratory rate reduction. This suggests that using the HFNO for early respiratory support and early optimization of postoperative respiratory function could be a strategy in this particular group of patients. ROX index, defined as the ratio of oxygen saturation (S_p_O_2_)/fraction of inspired oxygen (F_I_O_2_), is easily derived from commonly recorded variables measured in a non-invasive manner, and it is an early predictor of failure of high-flow nasal cannula (HFNC) (44).

The PRISM, a pragmatic multicenter randomized clinical trial in ~70 hospitals across five countries, is the largest study about noninvasive respiratory support after surgery, in which 4,806 patients were enrolled, and 4,793 were included in the final analysis (45). Of these, 2,396 were in the CPAP group and 2,397 in the control group. The primary composite outcome (pneumonia, endotracheal re-intubation or death within 30 days after randomization) occurred in 195/2,396 (8.1%) patients in the intervention group compared to 197/2,397 (8.2%) patients in the usual care group (OR 1.01 [0.81–1.24]; *p* = 0.95) showing that prophylactic CPAP did not reduce these complications after major abdominal surgery.

Recently, Abrard et al. reported the results of a postoperative prophylactic intermittent noninvasive respiratory support vs. usual postoperative care in cardiac or thoracic surgery of patients at high risk of PPC (46). They allocated 125 patients to prophylactic NRS and 128 to usual care. No difference was found in the incidence of postoperative acute respiratory failure between groups (NRS 24.0% vs. usual care 35%; OR 0.97 [0.90–1.04]; *p* = 0.54). Furthermore, prophylactic NRS was difficult to implement because of low patient compliance. Therefore, for noninvasive ventilation support during the postoperative period the level of evidence remains low. A different setting is the ICU, where a recent systematic review and network meta-analysis by Boscolo et al. found that NRS reduced the rate of post-extubation respiratory failure and ventilator associated pneumonia (VAP) compared with conventional oxygen therapy (COT) in high-risk and post-surgical patients, but not in the low-risk subgroups” (47).

### 4.1 NRS limitation and complication

One established complication of NRS could be barotrauma (48). Barotrauma includes, as a consequence, pneumothorax, pneumomediastinum and subcutaneous emphysema (49). A patient’s vigorous breathing can be the trigger by increasing transpulmonary pressure gradient across lung regions and global and regional strain (50), also known as the phenomenon of patient self-inflicted lung injury (P-SILI). Therefore, performing NRS in an appropriate setting and controlling ventilatory pressure support through skilled personnel is fundamental to avoid this complication. The risk of barotrauma associated with different types of NRS in the preoperative setting is low. However, a recent study in patients with COVID-19 has shown that CPAP/PSV increased the risk of barotrauma compared with HFNO (51).

## 5 Conclusion

In the last few years, the application of perioperative NRS has been expanding with increasing recognition of its potential role in the preoperative setting in high-risk patients for PPC or as a physiological optimization to prolong patients’ desaturation after anesthesia induction for difficult airway management. Another important application is preoperative NRS use as a therapeutic device in those patients with pre-existing sleep apnea disorder. Recognizing that the potential impact of preoperative NRS seems to be relevant. Postoperative NRS application is more common and logistically easier for skilled nurses, physiotherapists, intensive care physicians, and in the sub-intensive or intensive care space.

## Author contributions

LV: Conceptualization, Supervision, Writing – original draft, Writing – review & editing. CD: Writing – original draft, Writing – review & editing. NC-A: Writing – original draft, Writing – review & editing. FT: Writing – original draft, Writing – review & editing. CF: Writing – original draft, Writing – review & editing. SM: Writing – original draft, Writing – review & editing.
